# β-Hydroxy-β-methylbutyrate (HMB) Counteracts Atrophy and Restores Circadian Rhythms in Myotubes

**DOI:** 10.3390/ijms27146189

**Published:** 2026-07-10

**Authors:** Meytal Cohen-Or, Nava Chapnik, Natalie Avital-Cohen, Oren Froy

**Affiliations:** Institute of Biochemistry, Food Science and Nutrition, Robert H. Smith Faculty of Agriculture, Food and Environment, The Hebrew University of Jerusalem, Rehovot 76100, Israel; meytal.cohenor@mail.huji.ac.il (M.C.-O.); nava.chapnik@mail.huji.ac.il (N.C.); natalie.avital@mail.huji.ac.il (N.A.-C.)

**Keywords:** HMB, mTOR, clock, circadian, atrophy, metabolism, muscle, P70S6K

## Abstract

β-hydroxy-β-methylbutyrate (HMB), a bioactive metabolite of leucine, is widely recognized for its anabolic and anti-catabolic effects in skeletal muscle. However, the molecular mechanisms underlying these effects, particularly in relation to circadian regulation, remain incompletely understood. Here, we investigated the impact of HMB on dexamethasone-induced muscle atrophy in C2C12 myotubes, with a focus on anabolic signaling and circadian clock regulation. C2C12 myotubes were treated with HMB or HMB after dexamethasone-induced atrophy. HMB treatment significantly improved cell viability, surface area and fiber diameter by reducing expression of CBL-B, MuRF1 and Atrogin1, key mediators of muscle proteolysis, and increasing myogenin expression compared with atrophic conditions. While HMB did not activate AKT or mTOR, it robustly increased phosphorylation of P70S6K and S6 through a phospholipase D (PLD)-dependent mechanism. HMB restored disrupted circadian clock gene expression induced by dexamethasone, including normalization of expression patterns. HMB also enhanced circadian rhythmic amplitude and advanced phase timing, indicating improved clock robustness. These findings identify circadian regulation as a novel target of HMB action and demonstrate that HMB preserves muscle homeostasis through coordinated modulation of anabolic signaling and intrinsic circadian machinery. This study provides mechanistic insight into how HMB protects against muscle atrophy and highlights circadian regulation as an important contributor to skeletal muscle health.

## 1. Introduction

Skeletal muscle is a highly plastic tissue that continuously adapts to nutritional, hormonal and mechanical cues. Maintenance of muscle mass depends on a delicate balance between protein synthesis and degradation, and disruption of this balance leads to muscle atrophy in conditions such as aging, glucocorticoid exposure, cancer cachexia and metabolic disease [1]. Among the molecular pathways governing muscle mass, the ubiquitin–proteasome system and anabolic signaling cascades play central roles in determining muscle fate, where CBL-C, MuRF1 and Atrogin1 are key players [2,3].

β-hydroxy-β-methylbutyrate (HMB), a metabolite of leucine, has been shown to promote muscle hypertrophy, suppress protein degradation and enhance muscle recovery under catabolic conditions [4,5]. Mechanistically, HMB has been reported to stimulate protein synthesis and inhibit proteolysis by modulating signaling pathways involved in muscle turnover. While some studies indicate that HMB activates protein kinase B–mammalian target of rapamycin (AKT-mTOR) signaling, others demonstrate that its anabolic effects can occur independently of AKT, implicating alternative pathways, such as phospholipase D (PLD) and phosphatidic acid signaling [6].

Recent evidence suggests that skeletal muscle metabolism is also under strong circadian control. The intrinsic muscle clock, composed of core transcriptional regulators including brain and muscle Arnt-like protein-1 (BMAL1), circadian locomotor output cycles kaput (CLOCK), periods (PERs), cryptochrome (CRYs), reverse Erb (REV-ERBs) and retinoic acid-related orphan receptors (RORs), orchestrates daily oscillations in glucose metabolism, mitochondrial function and protein turnover [7,8]. Disruption of this clock leads to impaired metabolic flexibility, insulin resistance and muscle weakness. Notably, muscle-specific deletion of *Bmal1* results in severe muscle degeneration, mitochondrial dysfunction and premature aging [9,10]. Circadian dysregulation has also been implicated in muscle atrophy and aging [11]. Reduced circadian amplitude and altered phase timing are hallmarks of metabolic dysfunction and sarcopenia and are associated with impaired substrate utilization and diminished anabolic responsiveness [12]. Glucocorticoids, commonly used to induce muscle atrophy, directly interfere with circadian gene expression and suppress clock amplitude, further contributing to muscle wasting [8].

Despite growing recognition of the importance of circadian regulation in muscle physiology, little is known about whether anabolic interventions, such as HMB, can modulate the muscle clock. Preliminary work from our group demonstrated that HMB alters circadian gene expression and activates PLD-dependent anabolic signaling in myotubes and muscle tissue [13,14]. However, whether these effects contribute to protection against muscle atrophy and how circadian regulation integrates with anabolic signaling remains unresolved. In the present study, we investigated the effects of HMB on dexamethasone-induced muscle atrophy in C2C12 myotubes, focusing on anabolic signaling, ubiquitin-mediated proteolysis and circadian clock regulation. We demonstrated that HMB counteracts atrophy by suppressing proteolytic signaling, activating PLD-dependent anabolic pathways and restoring circadian clock gene expression and rhythmicity. These findings identify circadian regulation as a novel mechanism underlying the protective effects of HMB on skeletal muscle and provide new insight into its therapeutic potential for muscle-wasting conditions.

## 2. Results

To study the effect of HMB on the process of cell atrophy, C2C12 cells were divided into four groups: Control, Control + HMB, Atrophy, and Atrophy + HMB. The Control group was maintained in standard medium supplemented with DMSO at the same concentration used in HMB-treated groups. To synchronize circadian timing, the Control and Control + HMB groups were additionally treated with dexamethasone for 1 h. The Atrophy and Atrophy + HMB groups were treated with dexamethasone for 48 h to induce atrophy, in the absence or presence of HMB, respectively. Cells from all groups were collected at 0, 6, 12, 18, and 24 h for subsequent analyses.

### 2.1. The Effect of HMB on Muscle Growth in C2C12 Cells Undergoing Atrophy

**Figure 1:**
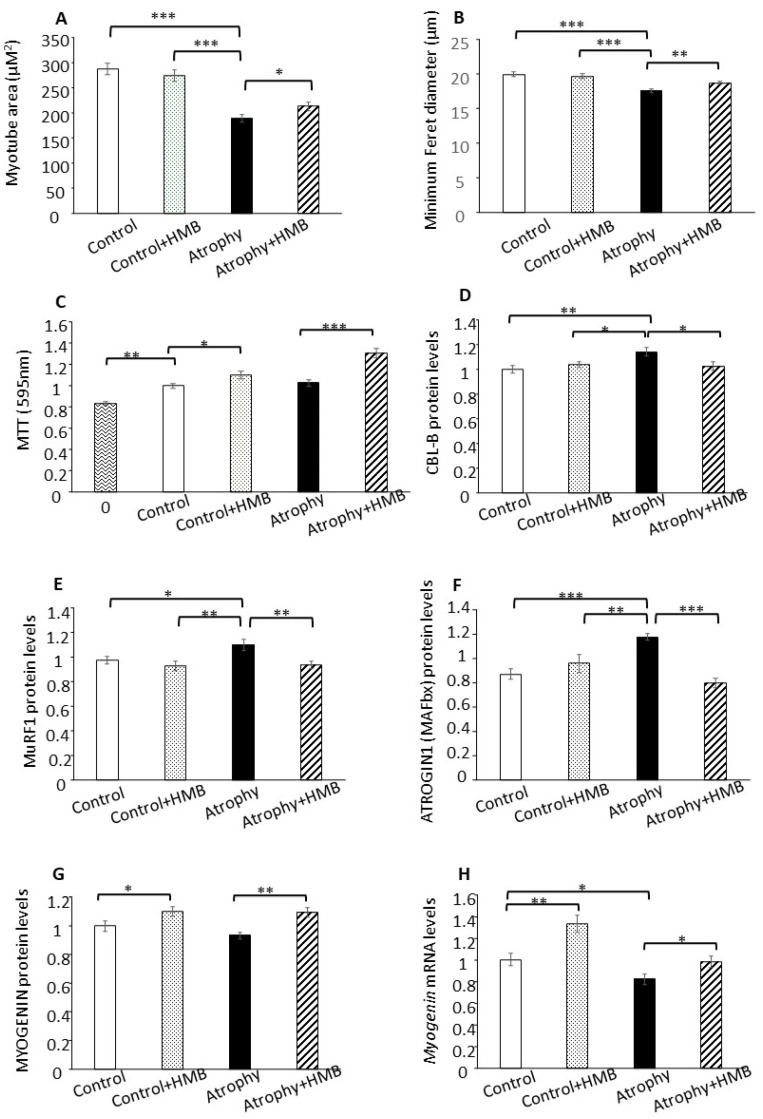
The effect of HMB on muscle growth in C2C12 cells undergoing atrophy. (**A**) Myotube fiber diameter. (**B**) Myotube surface area. (**C**) MTT-cell viability. (**D**) Daily CBL-B protein levels. (**E**) Daily MuRF1 protein levels. (**F**) Daily Atrogin1 protein levels. (**G**) Daily myogenin protein levels. (**H**) Daily *myogenin* mRNA levels. Daily values represent the mean of all time points collected across the 24 h circadian cycle. Three independent experiments were performed. Data are presented as means ± SEM. * *p* < 0.05, ** *p* < 0.01, and *** *p* < 0.0001.

Dexamethasone treatment significantly reduced myotube fiber diameter and surface area compared with control conditions, confirming the induction of atrophy in our experimental model. HMB led to an increase in myotube fiber diameter and surface area (*p* < 0.05) (Figure 1A,B). We also measured cell viability as a result of atrophy induction. The MTT levels showed a significant increase in the Control group compared with zero time (medium only) (*p* < 0.01), in the Control + HMB group compared with the Control group (*p* < 0.05), and in the Atrophy + HMB group compared with the Atrophy group (*p* < 0.0001) (Figure 1C). This suggests that HMB led to higher cell viability, increased proliferation, or improved cellular metabolism. To test whether atrophy was indeed induced, we measured protein levels of CBL-B, a critical E3 ubiquitin ligase that promotes skeletal muscle breakdown, MuRF1 and Atrogin1, two classical markers of muscle atrophy. We found significantly increased levels of CBL-B, MuRF1 and Atrogin1 in the Atrophy group compared with the Control group (*p* < 0.01) and reduced levels in the Atrophy + HMB group (*p* < 0.05) (Figure 1D–F; Supplementary Figure S1). The MYOGENIN protein levels showed significantly increased levels in the Control + HMB group compared with the Control group (*p* < 0.05). The Atrophy + HMB group also showed significantly increased MYOGENIN levels compared with the Atrophy group (*p* < 0.01) (Figure 1G; Supplementary Figure S1). The *myogenin* mRNA levels showed a similar trend between the groups: The Control + HMB group showed significantly increased levels compared with the Control group (*p* < 0.01), and the Atrophy + HMB group showed significantly increased levels compared with the Atrophy group (*p* < 0.05) as well. Additionally, the Atrophy group presented decreased myogenin levels compared with the Control group (*p* < 0.05) (Figure 1H). CBL-B and myogenin protein levels exhibited temporal oscillations in C2C12 myotubes across the experimental time course. Dexamethasone-induced atrophy altered these patterns by increasing the atrophy-associated protein CBL-B and reducing myogenin levels, while HMB treatment partially restored a control-like oscillatory profile (Supplementary Figure S2).

### 2.2. The Effect of Atrophy and HMB on Myotube Anabolism

**Figure 2:**
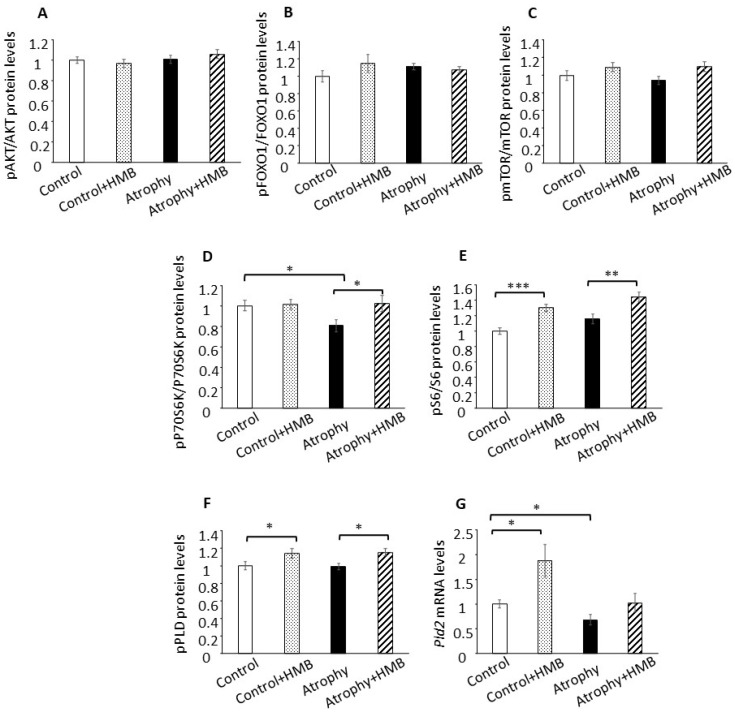
The effect of atrophy and HMB on myotube anabolism. (**A**) Daily pAKT/AKT protein levels. (**B**) Daily pFOXO1/FOXO1 protein levels. (**C**) Daily pmTOR/mTOR protein levels. (**D**) Daily pP70S6K/P70S6K protein levels. (**E**) Daily pS6/S6 protein levels. (**F**) Daily pPLD protein levels. (**G**) Daily *Pld2* mRNA levels. Daily values represent the mean of all time points collected across the 24 h circadian cycle. Three independent experiments were performed. Data are presented as means ± SEM. * *p* < 0.05, ** *p* < 0.01, and *** *p* < 0.0001.

To investigate the effect of atrophy on myotubes and whether HMB can counteract the induced damage, we examined the proteins in the muscle myogenesis pathway: AKT, FOXO1, mTOR, P70S6K, and S6 in their phosphorylated and unphosphorylated forms. pAKT/AKT, pFOXO1/FOXO1 and pmTOR/mTOR did not show a significant difference among the groups (*p* > 0.05) (Figure 2A–C; Supplementary Figure S3). In contrast, P70S6K and S6, the downstream proteins in the mTOR pathway, showed altered phosphorylated to unphosphorylated ratios (Figure 2D,E; Supplementary Figure S3). The pP70S6K/P70S6K ratio was significantly lower in the Atrophy group compared with the Control group (*p* < 0.05) and significantly higher in the Atrophy + HMB group compared with the Atrophy group (*p* < 0.05) (Figure 2D). Similarly, the pS6/S6 ratio was significantly higher in the Atrophy + HMB group compared with the Atrophy group (*p* < 0.01) (Figure 2E). As we recently showed, HMB induces myogenesis independently of AKT and mTOR, and the effect of P70S6K and S6 is mediated through PLD [13]. The pPLD protein, which leads to increased pP70S6K levels, showed similar results to those seen with pP70S6K and pS6. The Control + HMB group showed a significant increase in protein levels compared with the Control group (*p* < 0.05), and the Atrophy + HMB group showed a significant increase compared with the Atrophy group (*p* < 0.05) (Figure 2F; Supplementary Figure S1). *Pld2* mRNA levels presented significantly higher levels in the Control + HMB group compared with the Control group (*p* < 0.05) and significantly higher levels in the Control group compared with the Atrophy group (*p* < 0.05) (Figure 2G). Key components of the AKT–mTOR anabolic signaling pathway (AKT, mTOR, P70S6K, and S6) and the catabolic regulator FOXO1 showed rhythmic fluctuations over time. Atrophy suppressed anabolic signaling and enhanced FOXO1 activity, whereas HMB treatment increased phosphorylation of anabolic proteins and attenuated catabolic signaling (Supplementary Figure S4).

### 2.3. The Effect of Atrophy and HMB on the Circadian Clock Levels in Myotubes

**Figure 3:**
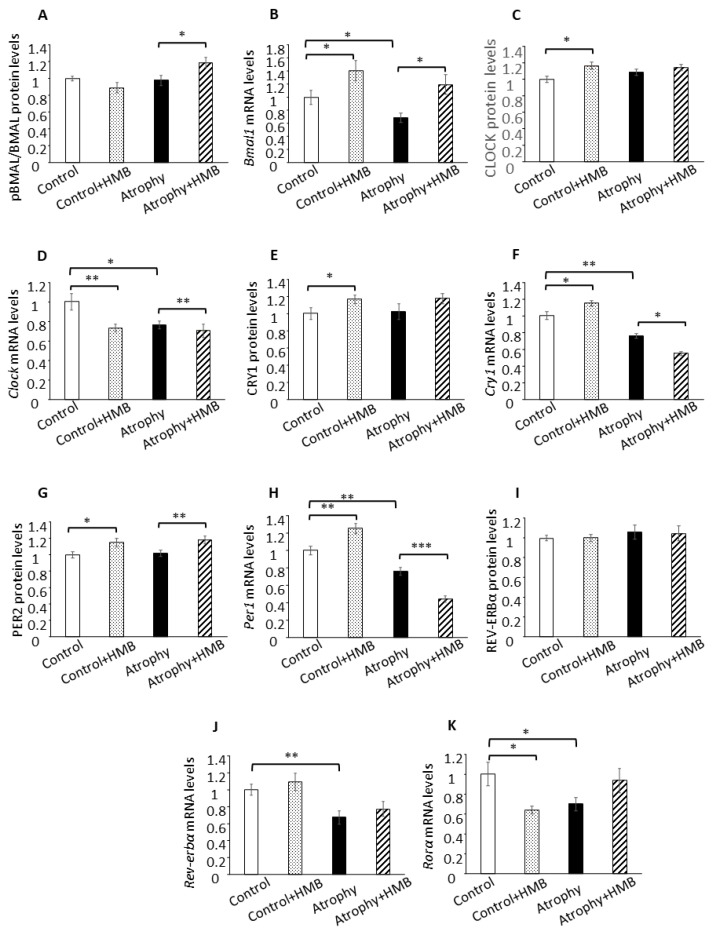
The effect of atrophy and HMB on the circadian clock levels in myotubes. (**A**) Daily pBMAL1/BMAL1 protein. (**B**) Daily *Bmal1* mRNA. (**C**) Daily CLOCK protein. (**D**) Daily *Clock* mRNA. (**E**) Daily CRY1 protein. (**F**) Daily *Cry1* mRNA. (**G**) Daily PER2 protein. (**H**) Daily *Per1* mRNA. (**I**) Daily REV-ERBα protein. (**J**) Daily *Rev-erbα* mRNA. (**K**) Daily *Rorα* mRNA. Daily values represent the means of all time points collected across the 24 h circadian cycle. Three independent experiments were performed. Data are presented as means ± SEM. * *p* < 0.05; ** *p* < 0.01, and *** *p* < 0.001.

We have previously shown that HMB alters circadian rhythms in cultured myotubes [13]. Therefore, we examined the effect of HMB on circadian clock gene expression in atrophy-induced myotubes in the presence or absence of HMB. The pBMAL1/BMAL1 ratio presented significantly higher levels in the Atrophy + HMB group compared with the Atrophy group (*p* < 0.05) (Figure 3A; Supplementary Figure S5). *Bmal1* mRNA showed significantly increased levels in the Control + HMB group compared with the Control group (*p* < 0.05) and a significant increase in the Atrophy + HMB group compared with the Atrophy group (*p* < 0.05). A significant decrease in the *Bmal1* mRNA levels was present in the Atrophy group compared with the Control group (*p* < 0.05) (Figure 3B). The CLOCK protein presented significantly increased levels in the Control + HMB group compared with the Control group (*p* < 0.05) (Figure 3C; Supplementary Figure S5). The *Clock* mRNA levels presented a decrease in the Control + HMB (*p* < 0.01) and Atrophy groups compared with the Control group (*p* < 0.05) and in the Atrophy + HMB group compared with the Atrophy group (*p* < 0.01) (Figure 3D).

CRY1 protein levels presented significantly increased levels in the Control + HMB group compared with the Control group (*p* < 0.05). No differences were shown between the Atrophy and the Atrophy + HMB groups (*p* > 0.05) (Figure 3E; Supplementary Figure S5). *Cry1* mRNA levels presented a significant increase in the Control + HMB group compared with the Control group (*p* < 0.05) and a significant decrease in the Atrophy + HMB group compared with the Atrophy group (*p* < 0.05). The Atrophy group showed a significant decrease in *Cry1* mRNA levels compared with the Control group (*p* < 0.01) (Figure 3F). PER2 protein levels presented a significant increase in the Control + HMB compared with the Control group (*p* < 0.05). The Atrophy + HMB group also showed a significant increase compared with the Atrophy group (*p* < 0.01) (Figure 3G; Supplementary Figure S5). *Per1* mRNA levels presented a significant increase in the Control + HMB group compared with the Control levels (*p* < 0.01), a significant decrease in the Atrophy + HMB group compared with the Atrophy group (*p* < 0.0001) and a significant decrease in the Atrophy group compared with the Control group (*p* < 0.01) (Figure 3H). The REV-ERBα protein levels did not show a significant difference among the groups (*p* > 0.05) (Figure 3I; Supplementary Figure S5). The *Rev-erbα* mRNA levels showed a significant decrease in the Atrophy group compared with the Control group (*p* < 0.01) (Figure 3J). *Rorα* mRNA levels presented a significant decrease in the Control + HMB group compared with the Control group (*p* < 0.05) and a significant decrease in the Atrophy group compared with the Control group (*p* < 0.05) (Figure 3K).

### 2.4. The Effect of Atrophy and HMB on the Circadian Clock Oscillation in Myotubes

While analysis of individual clock gene expression provides important information about molecular changes, circadian clock function is defined not only by expression levels but also by rhythmic properties, such as phase and amplitude. These parameters reflect the temporal coordination and robustness of the circadian system and are critical for proper regulation of metabolic and anabolic processes in skeletal muscle. Since dexamethasone is known to disrupt circadian rhythmicity and HMB has been shown to modulate clock gene expression, we next examined whether atrophy and HMB treatment alter the oscillatory patterns of core circadian genes, including their phase and amplitude, in C2C12 myotubes.

**Figure 4:**
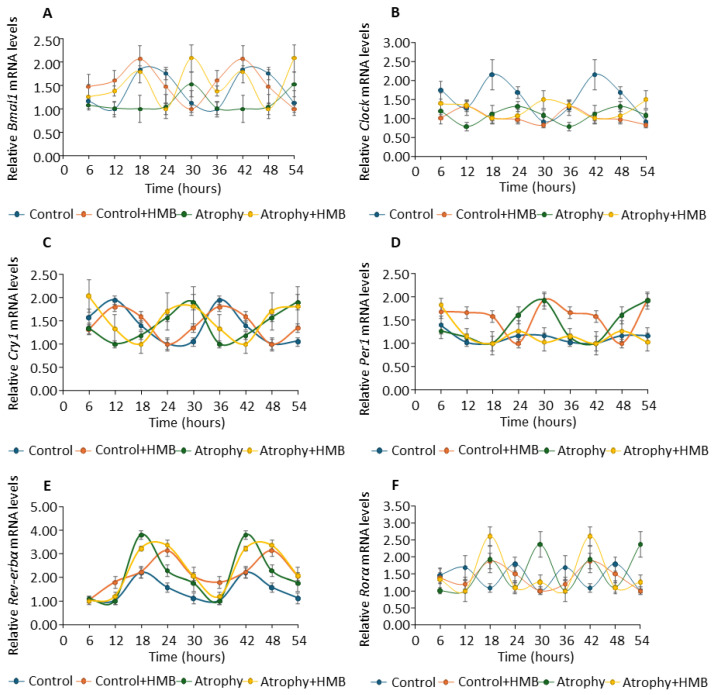
The effect of atrophy and HMB on the circadian clock oscillation in myotubes. (**A**) *Bmal1* mRNA. (**B**) *Clock* mRNA. (**C**) *Cry1* mRNA. (**D**) *Per1* mRNA. (**E**) *Rev-erbα* mRNA. (**F**) *Rorα* mRNA. Circadian rhythmicity and time-dependent effects were analyzed using one-way ANOVA across time points. Circadian rhythmicity (amplitude, phase, and mesor) was further analyzed using CircWave software (version 1.4). Three independent experiments were performed.

Bmal1 showed a phase advance and high amplitude in the Control + HMB group compared with the Control group and in the Atrophy + HMB group compared with the Atrophy group (Figure 4A). The relative clock mRNA levels showed decreased amplitude and phase advance in the Control + HMB group compared with the Control group. The Atrophy + HMB group exhibited a phase advance and higher amplitude compared with the Atrophy group (Figure 4B). The relative Cry1 mRNA showed no significant change in phase and amplitude between the Control + HMB and the Control groups, and between the Atrophy + HMB and the Atrophy groups. Both atrophy groups exhibited phase delay (Figure 4C). Relative Per1 mRNA levels presented a high amplitude in the Control + HMB group compared with the Control group and a low amplitude in the Atrophy + HMB group compared with the Atrophy group. No significant phase change was present in all groups (Figure 4D). The relative Rev-erbα mRNA levels showed a phase delay in the Control + HMB group compared with the Control group and in the Atrophy + HMB group compared with the Atrophy group. The Control + HMB group presented a high amplitude compared with the Control group (Figure 4E). The relative Rorα mRNA levels showed a phase delay in the Control + HMB group compared with the Control group. No differences in amplitude were seen between these groups (Figure 4F).

## 3. Discussion

In the present study, we investigated the ability of HMB to counteract dexamethasone-induced muscle atrophy in C2C12 myotubes, with a particular focus on anabolic signaling, ubiquitin-mediated degradation and circadian clock regulation. Inclusion of morphometric analysis, as well as atrophy markers, further validated the robustness of the dexamethasone-induced atrophy model used in this study. Our results demonstrate that HMB partially counteracts atrophy-induced impairments in cell viability, morphometric measurements and markers, restores myogenic signaling, and modulates circadian clock gene expression, highlighting the multifaceted protective role of HMB in skeletal muscle. The HMB concentration was selected based on our previous work and the published literature [13,15,16], demonstrating that 50 μM of HMB effectively activates anabolic signaling and modulates circadian gene expression in C2C12 myotubes without inducing cytotoxic effects. Specifically, our prior study demonstrated that this concentration activates phospholipase D-dependent signaling and alters circadian rhythms in myotubes, supporting its physiological relevance [13]. This concentration is also within the range commonly used in vitro to model physiologically relevant effects of HMB.

The results demonstrate that HMB attenuates dexamethasone-induced muscle atrophy and restores myogenic signaling. Dexamethasone treatment significantly increased expression of CBL-B, a muscle-specific E3 ubiquitin ligase known to promote proteolysis and inhibit insulin/IGF-1 signaling, as well as MuRF1 and Atrogin1, two canonical atrogenes that mediate myofibrillar protein degradation via the ubiquitin–proteasome system [1,2,3]. The observed increase in CBL-B in the atrophy group is consistent with previous reports demonstrating its role in glucocorticoid-induced muscle wasting. Similarly, the upregulation of MuRF1 and Atrogin1 further supports activation of a coordinated proteolytic program under dexamethasone treatment, contributing to muscle protein breakdown. HMB supplementation significantly attenuated these effects, increasing cell viability and reducing CBL-B expression. Importantly, HMB also blunted the induction of MuRF1 and Atrogin1, suggesting a broader inhibitory effect on atrophy-related ubiquitin ligases. These findings are consistent with previous studies showing that HMB suppresses proteasome-mediated protein degradation and preserves muscle mass under catabolic conditions [4]. The ability of HMB to blunt CBL-B induction suggests a direct effect on ubiquitin–proteasome signaling, which may underlie its anti-atrophic properties, potentially through coordinated suppression of multiple E3 ligases involved in muscle wasting.

In parallel, HMB significantly increased myogenin protein and mRNA levels under both basal and atrophic conditions. Myogenin is essential for myoblast differentiation and muscle regeneration, and its suppression is a hallmark of muscle wasting [17]. The increase in myogenin expression by HMB supports its role not only in preventing degradation but also in promoting myogenic differentiation and regeneration. Importantly, the increase in myogenin expression observed under basal conditions suggests that HMB does not merely counteract catabolic signaling but actively promotes anabolic and differentiation pathways. This effect may be mediated through activation of PLD-dependent signaling and increased phosphatidic acid production, which has been shown to stimulate myogenic transcription factors and enhance translational efficiency of muscle-specific genes [6,13,18]. Additionally, circadian regulators such as BMAL1 directly control myogenic gene expression [7,9], and restoration of circadian function by HMB may further contribute to enhanced myogenin expression, linking circadian regulation with muscle differentiation and maintenance.

Notably, several anabolic and circadian parameters were elevated by HMB even in the absence of atrophy. These findings suggest that HMB acts not only as an anti-catabolic agent but also as a metabolic modulator that enhances basal anabolic readiness. This effect may reflect improved translational efficiency, increased ribosomal activity and enhanced mitochondrial function, all of which are regulated by circadian clock components and nutrient-sensing pathways [9]. Such priming of anabolic pathways may improve the capacity of muscle cells to respond to physiological stimuli, including nutrient availability, mechanical load, or recovery from stress. This is consistent with previous studies demonstrating that HMB enhances protein synthesis and muscle cell differentiation even under non-catabolic conditions [5,19], supporting its role as a regulator of muscle homeostasis rather than solely a protective agent during atrophy.

HMB was also found to activate anabolic signaling downstream of mTOR via a PLD-dependent mechanism. Despite the central role of AKT and mTOR in muscle anabolism, we observed no significant changes in AKT or mTOR phosphorylation following HMB treatment. In contrast, the downstream effectors P70S6K and S6 were strongly affected. Dexamethasone reduced phosphorylation, while HMB restored their activation in atrophic myotubes. These findings are consistent with previous work demonstrating that HMB stimulates protein synthesis independently of AKT and mTOR activation [5,13,14,19]. Instead, HMB appears to act through PLD, leading to increased production of phosphatidic acid and subsequent activation of P70S6K [6]. The observed increase in PLD phosphorylation and *Pld2* mRNA expression in HMB-treated cells strongly supports this mechanism. This alternative anabolic pathway may be particularly relevant under catabolic conditions, where insulin/IGF-1 signaling is impaired. By bypassing canonical AKT-mTOR signaling, HMB may preserve protein synthesis even in the presence of glucocorticoids or metabolic stress. The apparent discrepancy between unchanged mTOR phosphorylation and increased phosphorylation of its downstream targets may reflect localized or transient activation of mTOR signaling that is not fully captured by bulk phosphorylation measurements. Alternatively, phosphatidic acid generated by PLD can directly enhance P70S6K activity or increase the sensitivity of downstream effectors to basal mTOR activity.

A major novel finding of this study is the ability of HMB to modulate circadian clock gene expression in atrophying myotubes. Dexamethasone markedly disrupted the expression of core clock components, including *Bmal1*, *Cry1*, *Per1* and *Rev-erbα*, consistent with previous reports linking glucocorticoid exposure and muscle wasting to circadian dysregulation [3,8]. These alterations reflect a breakdown of intrinsic muscle clock function, which is increasingly recognized as a critical regulator of muscle metabolism, growth and regeneration. HMB significantly restored BMAL1 expression and enhanced its rhythmic amplitude, suggesting normalization of circadian clock function. BMAL1 is a master regulator of skeletal muscle homeostasis, controlling mitochondrial biogenesis, oxidative metabolism, protein turnover and myogenic differentiation [8,9]. Muscle-specific deletion of *Bmal1* results in severe muscle weakness, reduced mitochondrial content, increased oxidative stress and premature aging phenotypes [9,10]. Moreover, BMAL1 has been shown to directly regulate genes involved in protein synthesis and degradation, including components of the ubiquitin–proteasome system, linking circadian control to muscle mass maintenance [3]. Restoration of BMAL1 signaling by HMB, therefore, represents a key mechanism by which muscle integrity may be preserved under catabolic conditions.

HMB also modulated the expression of other core clock components in a gene-specific manner. Although *Clock* mRNA levels were reduced, CLOCK protein abundance was increased, suggesting post-transcriptional regulation. Such discrepancies between transcript and protein levels are well documented in circadian biology and reflect tight control by translational efficiency, protein stability and post-translational modifications [20]. The CLOCK protein plays a critical role in maintaining transcriptional rhythmicity through heterodimerization with BMAL1, and its stabilization may contribute to the restoration of clock output despite reduced transcript levels. These findings suggest that HMB may influence circadian regulation at multiple levels, including translational control and protein stabilization, rather than acting solely at the transcriptional level. Nutrient-derived metabolites have been shown to regulate circadian protein stability and translation through modulation of cellular energy status, kinase signaling, and proteasomal degradation pathways [21]. Thus, HMB may enhance circadian clock function by improving the stability and functional activity of core clock proteins, even in cases where transcriptional changes are modest or delayed.

Alterations in *Cry1* and *Per1* expression further support a role for HMB in stabilizing circadian feedback loops. CRY and PER proteins act as transcriptional repressors within the clock machinery and are essential for maintaining oscillatory stability. Dysregulation of these components has been linked to muscle atrophy, insulin resistance, and impaired regeneration [3,22]. By partially normalizing their expression and rhythmicity, HMB may help restore temporal coordination between metabolic demand and anabolic signaling.

Notably, HMB partially restored rhythmic amplitude and advanced circadian phase in atrophic myotubes. Reduced circadian amplitude is a hallmark of metabolic dysfunction, aging and muscle wasting, and is associated with impaired substrate utilization, reduced insulin sensitivity and diminished anabolic responsiveness [23]. In humans, older and metabolically compromised individuals display disrupted day–night rhythmicity of core clock genes in skeletal muscle and impaired glucose oxidation rhythm in muscle, including blunted amplitude and altered phase [12,24]. Circadian disruption of muscle clock components has been linked to impaired metabolic flexibility and glucose intolerance [24]. Furthermore, core clock gene oscillations in skeletal muscle regulate pathways critical for metabolism, mitochondrial function and muscle homeostasis [7]. Dysregulation of the circadian timing system is also implicated in age-related muscle decline and sarcopenia [25], and broader circadian misalignment contributes to metabolic disease phenotypes [26]. Importantly, increased circadian amplitude reflects stronger oscillatory robustness and improved temporal coordination of gene expression, which enhances mitochondrial function, protein synthesis, and metabolic efficiency [27]. Similarly, phase advancement may allow earlier activation of anabolic and metabolic pathways, potentially improving the alignment between cellular energy production and anabolic demand. Restoration of circadian phase and amplitude by HMB may, therefore, improve the timing and efficiency of anabolic signaling, contributing to enhanced muscle maintenance and resistance to atrophic stimuli.

Collectively, these findings indicate that HMB not only acts as a metabolic modulator but also functions as a regulator of the skeletal muscle circadian clock. Through restoration of clock gene expression, enhancement of rhythmic amplitude and normalization of phase timing, HMB may protect against muscle atrophy by improving mitochondrial function, protein turnover and metabolic homeostasis. This circadian-based mechanism provides a novel framework for understanding the anabolic and protective effects of HMB in muscle-wasting conditions.

**Figure 5:**
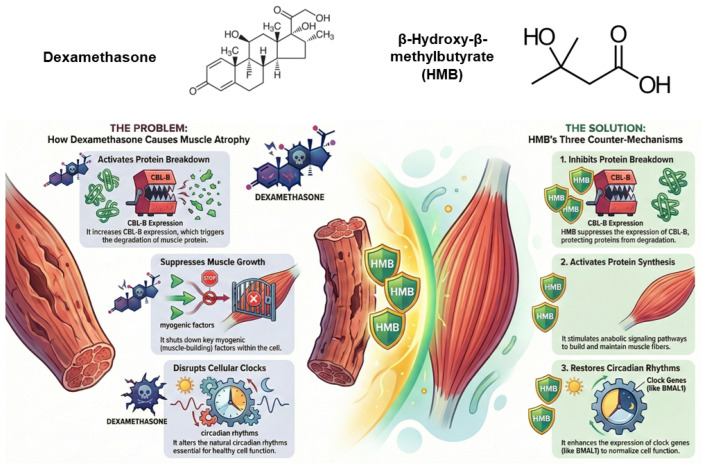
Schematic model of the effect of dexamethasone vs. HMB in myotubes. HMB counteracts dexamethasone-induced muscle atrophy by suppressing proteolytic signaling, activating PLD-dependent anabolic pathways and restoring circadian clock gene expression (generated via NotebookLM application).

Our data support a model in which HMB protects skeletal muscle through three complementary mechanisms: 1. Suppression of proteolysis via downregulation of CBL-B and attenuation of ubiquitin–proteasome activity; 2. activation of anabolic signaling mediated by PLD-dependent activation of P70S6K and S6 independently of AKT/mTOR; and 3. restoration of circadian rhythmicity, particularly through modulation of BMAL1 and associated clock genes (Figure 5). This integrated mechanism links nutrient sensing, circadian regulation and muscle protein turnover, providing a broader framework for understanding how HMB preserves muscle mass under catabolic conditions.

It is noteworthy that dexamethasone did not uniformly affect all markers examined in this study. While glucocorticoid-induced muscle atrophy is classically associated with activation of proteolytic pathways such as the ubiquitin–proteasome system (e.g., MuRF1 and Atrogin1), anabolic and myogenic markers do not necessarily respond in a uniform manner. In particular, myogenin primarily reflects differentiation and regenerative processes rather than acute catabolic signaling, which may explain its limited responsiveness under the present experimental conditions. Similarly, phosphorylation-dependent signaling proteins such as pS6/S6 and pPLD/PLD can exhibit transient or context-dependent changes that are influenced by treatment duration, cellular state, and circadian timing. Therefore, the partial effects observed across markers likely reflect the selective and pathway-specific nature of glucocorticoid-induced muscle atrophy rather than an absence of an atrophic response.

Although our findings demonstrate that HMB modulates both atrophy-related signaling pathways and circadian clock components, the present study does not establish a direct causal relationship between these processes. It remains unclear whether the protective effects of HMB on muscle atrophy are mediated through circadian regulation or whether these represent parallel, independently regulated mechanisms. Future studies employing genetic or pharmacological manipulation of core clock components (e.g., BMAL1 knockdown) will be required to determine the causal contribution of circadian modulation to the anti-atrophic effects of HMB.

Although this study provides mechanistic insight, it is limited by its in vitro design. First, additional classical atrophy markers, such as IRS-1, would strengthen model validation and should be included in future studies. Although we have shown that HMB can exert distinct effects independent of leucine, particularly through PLD-mediated pathways, future studies directly comparing leucine and HMB would be valuable to further distinguish their specific roles. Future in vivo studies are needed to validate these findings and to determine whether circadian modulation contributes to the clinical efficacy of HMB in aging, cachexia, or glucocorticoid-induced myopathy. Additionally, the precise molecular interaction between PLD signaling and circadian regulators warrants further investigation. In conclusion, HMB effectively counteracts dexamethasone-induced muscle atrophy by suppressing proteolytic signaling, activating PLD-dependent anabolic pathways and restoring circadian clock gene expression. These findings identify circadian regulation as a novel component of HMB action and support its therapeutic potential in conditions characterized by muscle wasting and circadian disruption.

## 4. Materials and Methods

### 4.1. Cell Culture and Treatments

C2C12 myoblasts were cultured in Dulbecco’s modified Eagle’s medium (DMEM) (Biological Industries, Beit HaEmek, Israel) supplemented with 10% bovine serum and 1% antibiotic solution (penicillin 10,000 U/mL and streptomycin 10 mg/mL) (Biological Industries, Beit HaEmek, Israel) and maintained at 37 °C in a humidified atmosphere containing 5% CO_2_. Myogenic differentiation was induced once cells reached confluence by replacing the growth medium with DMEM supplemented with 2% horse serum. The differentiation medium was refreshed daily for 48–72 h, during which myotube formation was achieved. After differentiation for 48 h, the control group was treated with DMSO, the Control + HMB group was treated with DMSO+ beta-hydroxy-beta-methylbutyrate (HMB) (Sigma, Rehovot, Israel) to a final concentration of 50 μM based on previous studies [13,15,16], the Atrophy group was treated with 1 µM dexamethasone and the Atrophy + HMB group was treated with 1 µM dexamethasone + 50 μM HMB. After 48 h, the Control group and Control + HMB group cells were synchronized with a 1 h pulse of 1 µM dexamethasone. Following 6 h of incubation, the cells were harvested in triplicate per treatment per time point every 6 h for 24 h. The 24 h time-course analysis was selected to capture circadian oscillations and temporal dynamics of gene and protein expression following synchronization. While endpoint measurements at 48 h post-treatment would provide static comparisons, they would not reflect rhythmic variations, which were central to the objectives of this study. Two biologically independent experiments were conducted on separate occasions, each including all four treatment groups and performed in triplicate per time point. Data from both experiments were pooled for statistical analysis.

### 4.2. Cell Viability Assay

Cell viability was assessed using the 3-(4,5-dimethylthiazol-2-yl)-2,5-diphenyltetrazolium bromide (MTT) assay, which reflects cellular metabolic activity through mitochondrial redox potential. Metabolically active cells reduce the water-soluble MTT reagent to insoluble purple formazan crystals. Following treatment with DMSO, DMSO + HMB, dexamethasone, or dexamethasone + HMB, the culture medium was removed, and cells were incubated with 50 μL of MTT solution (0.5 mg/mL in DMEM without phenol red) for 2 h at 37 °C in a humidified 5% CO_2_ atmosphere. The MTT solution was then discarded, and formazan crystals were solubilized in dimethyl sulfoxide (DMSO). The absorbance was measured at 595 nm using a microplate reader, and the results were expressed as relative optical density.

### 4.3. Immunofluorescent Staining

The cells were washed with PBS, then fixed with 4% paraformaldehyde (PFA) diluted in PBS for 20 min and washed two times with PBS. The cells were permeabilized with 0.5% Triton in PBS for 10 min and washed again with PBS two times. Subsequently, DAPI (1:1000, Sigma-Aldrich, Rehovot, Israel) and phalloidin (1:1000) (Abcam, Cambridge, UK), both diluted in PBS, were added, and the cells were incubated for 1 h at room temperature before imaging.

### 4.4. Myotube Morphometric Analysis

Myotube morphology was assessed by measuring myotube fiber diameter and surface area following treatments with an Andor BC43 confocal microscope (Oxford Instruments, Abingdon, UK). Myotube fiber diameter was estimated using the MinFeret diameter. Myotube fiber and myotube area were quantified using automated image analysis Fiji software ImageJ (version 1.54t). Where applicable, AI-based segmentation tools were used to enhance the accuracy and reproducibility of myotube boundary detection. At least 3 fields per condition were analyzed, and measurements were averaged per experiment.

### 4.5. Western Blot Analyses

Cells were lysed in 200 μL of lysis buffer (pH 7.8) containing 20 mM Tris, 145 mM NaCl, 5% glycerol, 1% Triton X-100, 50 nM phenylmethylsulfonyl fluoride (PMSF), 50 μM sodium fluoride, 10 μM sodium orthovanadate, 50 ng/mL aprotinin, 100 ng/mL leupeptin, and 0.8 μg/mL trypsin inhibitor (Sigma, Rehovot, Israel). Protein samples were separated on 10% SDS–polyacrylamide gels and transferred to nitrocellulose membranes using a semi-dry transfer system. Membranes were incubated with primary antibodies against BMAL1 and phosphorylated BMAL1, AKT, FOXO1 and pFOXO1, P70S6K and pP70S6K, S6 and pS6, CBL-B (Cell Signaling Technology, Beverly, MA, USA), ACTIN, pAKT, mTOR and pmTOR, MYOGENIN, CLOCK, CRY1, REV-ERBα, MuRF1, Atrogin1 (MAFbx) (Santa Cruz Biotechnology, Santa Cruz, CA, USA), pPLD (Invitrogen, Thermo Fisher Scientific, Fair Lawn, NJ, USA), PER2 (ABclonal Technology, Woburn, MA, USA), and HSP70 (Abcam, Cambridge, UK). After washing, membranes were incubated with horseradish peroxidase-conjugated secondary antibodies (Pierce, Rockford, IL, USA). ACTIN was used as a loading control and detected using an anti-mouse antibody (MP Biomedicals, Solon, OH, USA). Protein bands were visualized using enhanced chemiluminescence (Santa Cruz Biotechnology) and quantified by densitometry using Image Lab software (version 3.01) (Bio-Rad, Hercules, CA, USA). The results are expressed as arbitrary units relative to loading controls.

### 4.6. Total RNA Extraction and Quantitative Real-Time PCR

Total RNA was extracted using TRI Reagent (Sigma, Rehovot, Israel) according to the manufacturer’s protocol. RNA samples were treated with DNase I (RQ1 DNase, Promega, Madison, WI, USA) and reverse-transcribed using the qScript cDNA Synthesis Kit (Quanta BioSciences, Gaithersburg, MD, USA) with random hexamers. Quantitative real-time PCR was performed using gene-specific primers and the ABI Prism 7300 Sequence Detection System (Applied Biosystems, Foster City, CA, USA). The amplification conditions were 95 °C for 3 min, followed by 40 cycles of 95 °C for 10 s and 60 °C for 45 s. Gene expression levels were normalized to actin and calculated using the 2^−ΔΔCt^ method.

### 4.7. Statistical Analyses

All data are presented as means ± standard error (SEM). Statistical significance between groups was assessed using Student’s *t*-test or ANOVA and Tukey’s honestly significant difference (HSD) post hoc test, where appropriate. Circadian rhythmicity and time-dependent effects were analyzed using one-way ANOVA across time points. Circadian rhythmicity (amplitude, phase, and mesor) was further analyzed using CircWave software (version 1.4; Circadian Rhythm Laboratory, University of Groningen, The Netherlands) by applying harmonic regression or Fourier-curve fit analyses to biological data. A *p*-value of < 0.05 was considered statistically significant. Statistical analyses were performed using JMP software (version JMP Student Edition 18, SAS Institute, Cary, NC, USA).

## Data Availability

The datasets generated during and/or analyzed during the current study are available from the corresponding author upon reasonable request.

## Supplementary Materials

The following supporting information can be downloaded at https://www.mdpi.com/article/10.3390/ijms27146189/s1.

## Abbreviations

The following abbreviations are used in this manuscript:

AKT	Protein kinase B
BMAL1	Brain and muscle Arnt-like protein-1
CLOCK	Circadian locomotor output cycles kaput
CRY	Cryptochrome
HMB	β-hydroxy-β-methyl butyrate
mTOR	Mammalian target of rapamycin
PER	Period
PLD	Phospholipase D
REV-ERB	Reverse Erb
ROR	Retinoic acid-related orphan receptor
