# Supplementary material for: β-Hydroxy-β-methylbutyrate (HMB) Counteracts Atrophy and Restores Circadian Rhythms in Myotubes
Source: Int J Mol Sci. 2026 Jul 10;27(14):6189. doi: 10.3390/ijms27146189 (PMC13410136; doi:10.3390/ijms27146189)
Supplement: Supplementary file 1 [file ijms-27-06189-s001.zip › ijms-4307976-supplementary.pdf]

Supplementary Figure S1

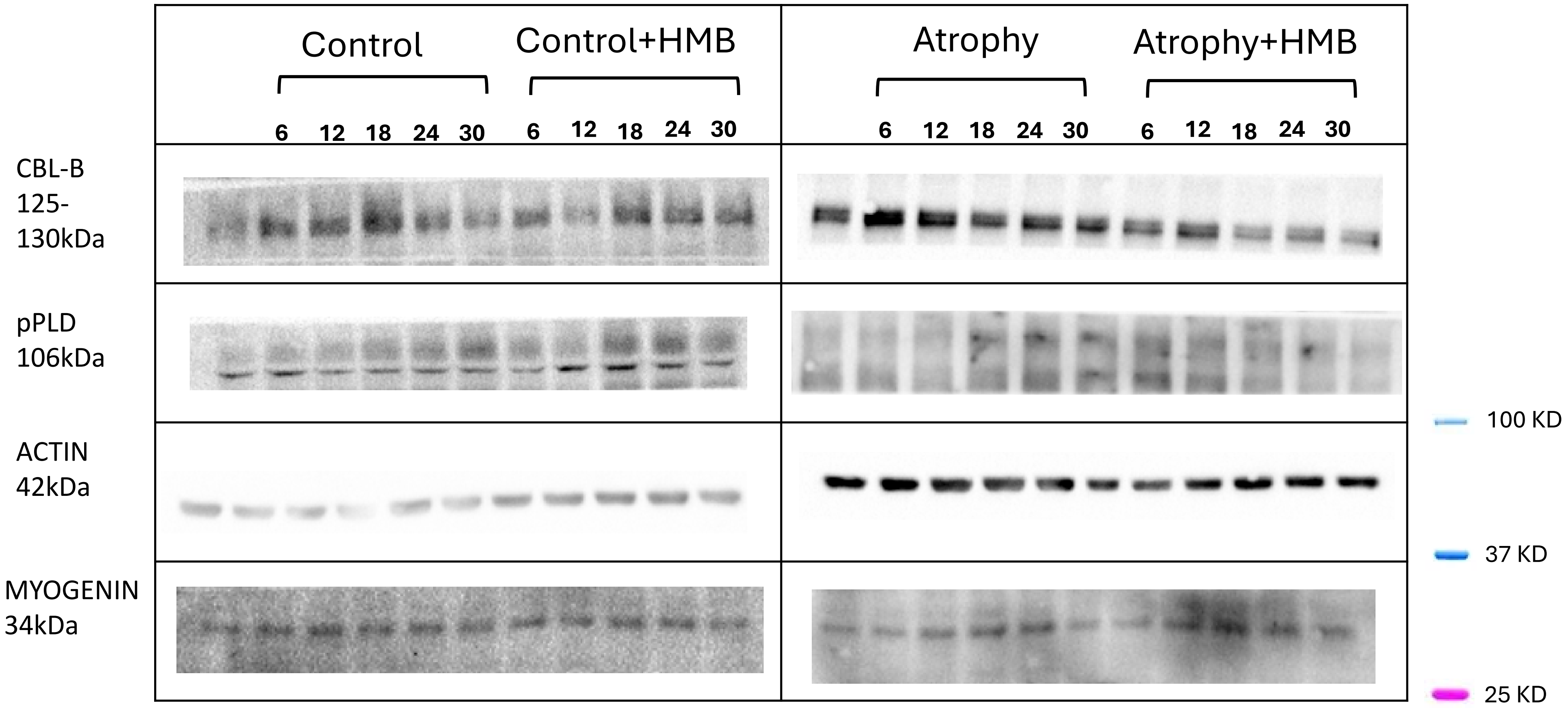

Supplementary figure S1: Western blot proteins analysis of the effect of HMB on muscle growth in C2C12 cells undergoing atrophy.

Supplementary Figure S2

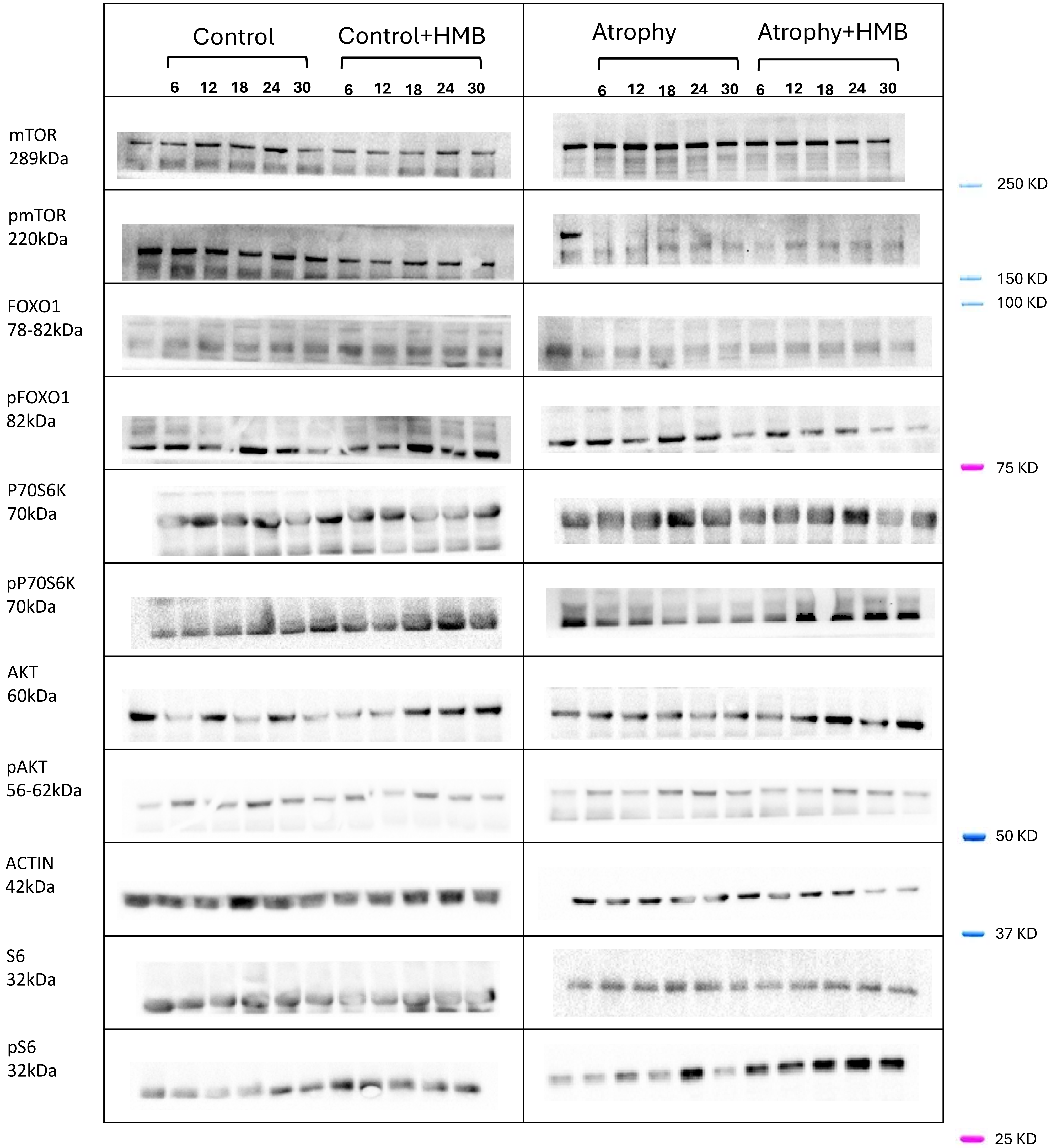

Supplementary figure S2: Western blot proteins analysis of atrophy and HMB supplementation on myotube anabolism.

Supplementary Figure S3

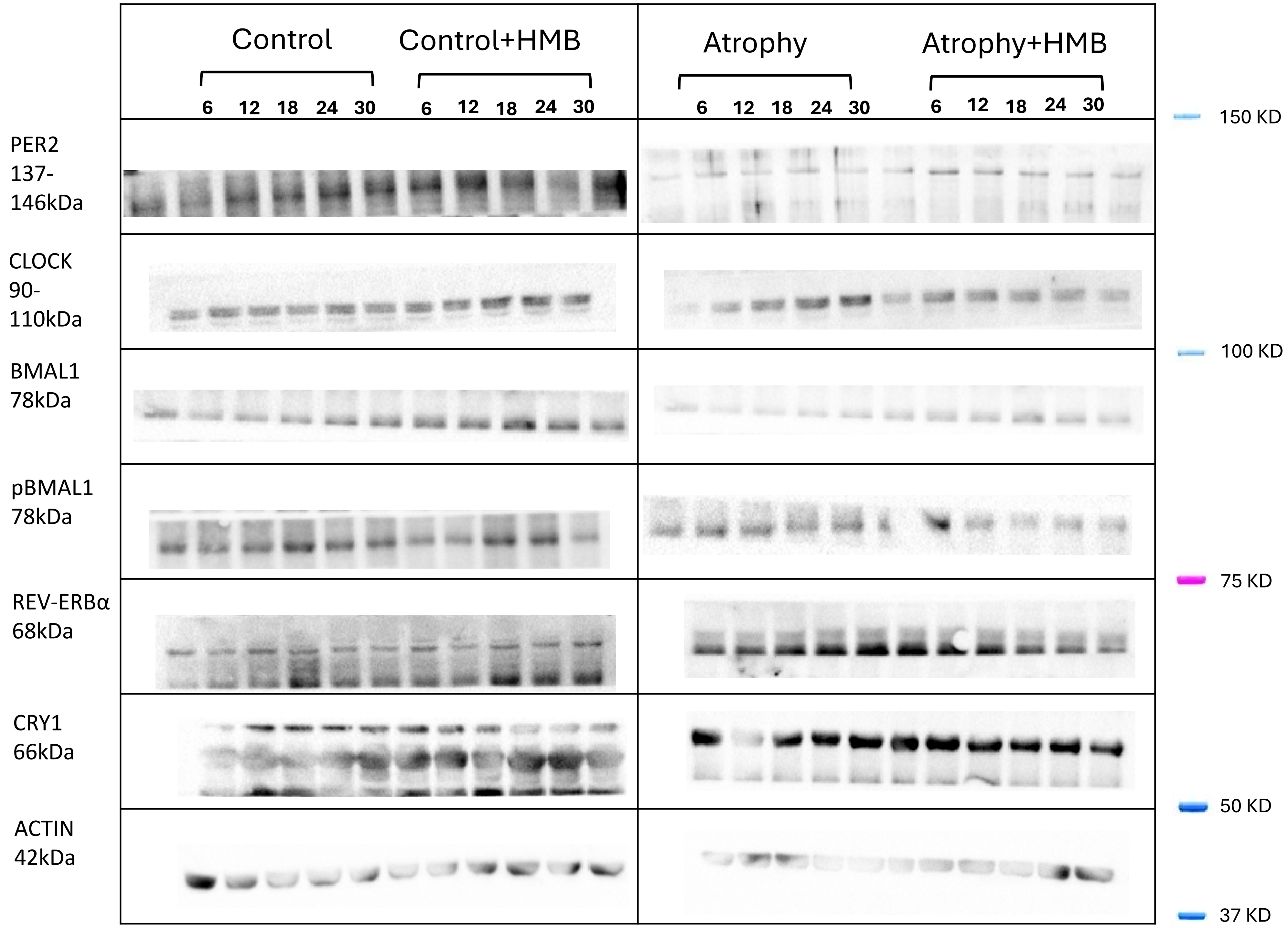

Supplementary figure S3: Western blot proteins analysis of the effect of atrophy and HMB supplementation on the circadian clock in myotubes.

Supplementary Figure S4

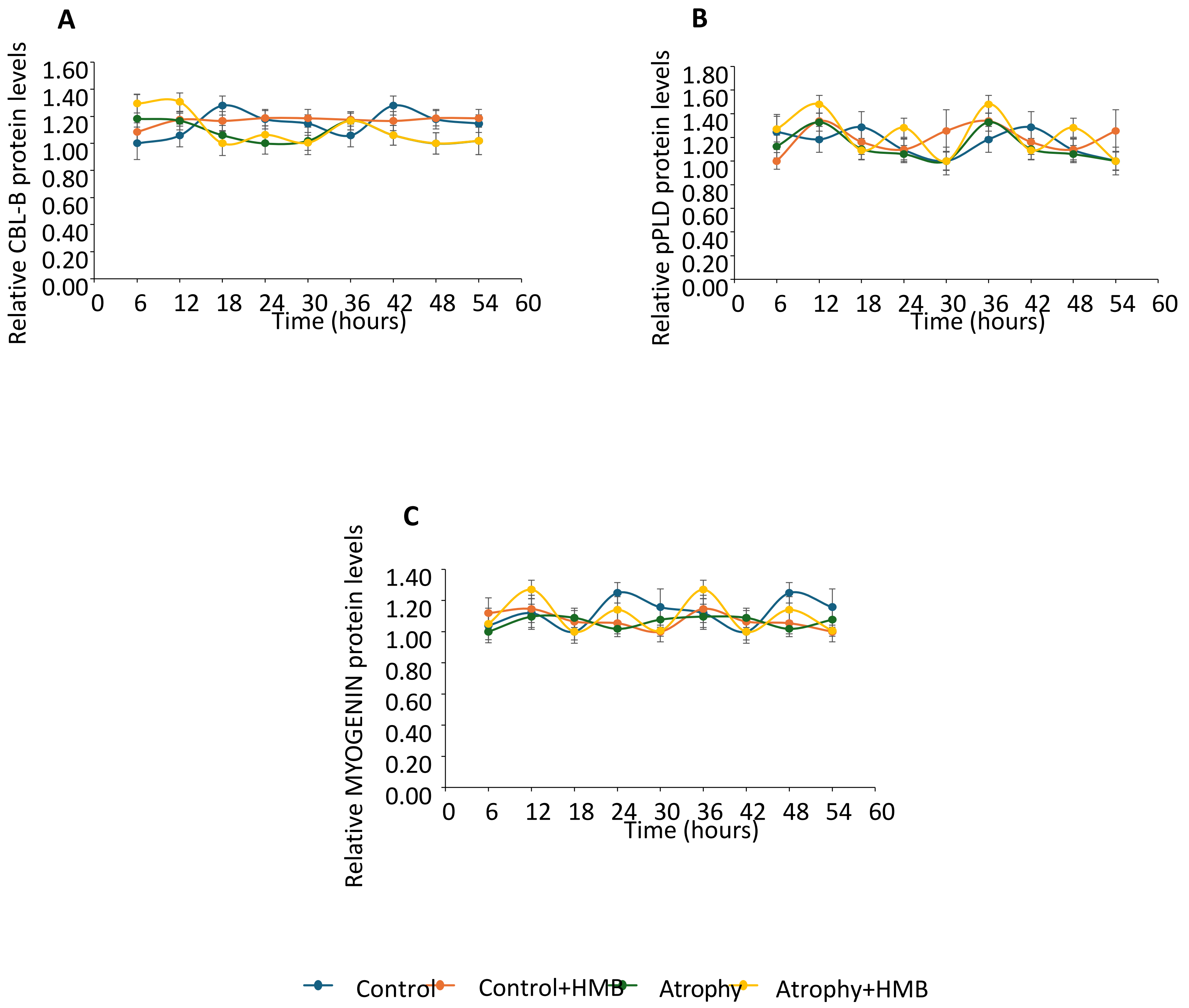

Supplementary figure S4: Oscillation of atrophy and growth proteins in C2C12 cells undergoing atrophy.

Supplementary Figure S5

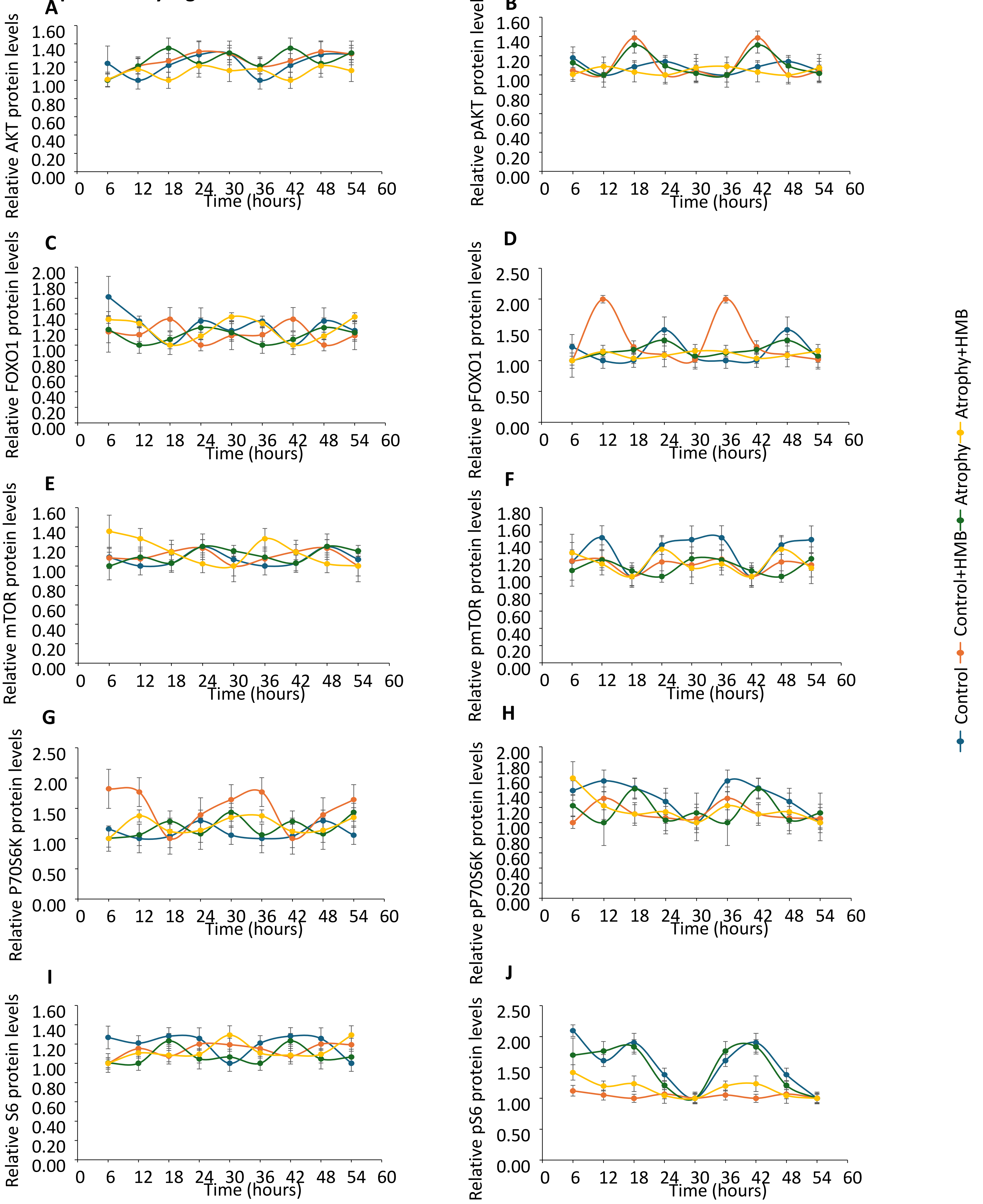

Supplementary figure S5: Anabolic proteins oscillations.

Supplementary Figure S6

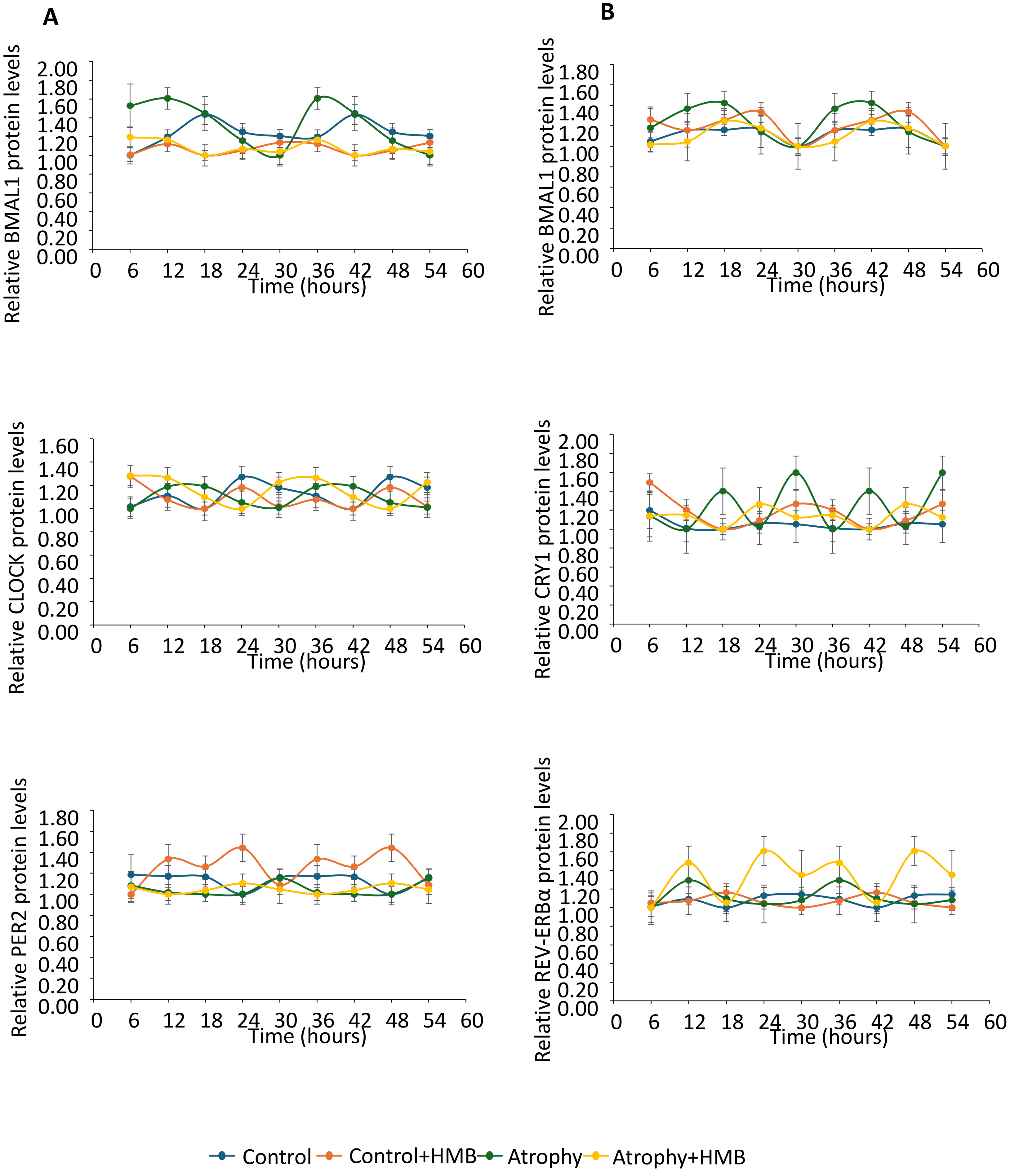

Supplementary figure S6: Circadian clock oscillations in C2C12 cells under different treatments.
